# CdSe/TiO_2 _core-shell nanoparticles produced in AOT reverse micelles: applications in pollutant photodegradation using visible light

**DOI:** 10.1186/1556-276X-6-426

**Published:** 2011-06-15

**Authors:** Arlindo M Fontes Garcia, Marisa SF Fernandes, Paulo JG Coutinho

**Affiliations:** 1 Centre of Physics (CFUM), University of Minho, Campus de Gualtar, 4710-057 Braga, Portugal

## Abstract

CdSe quantum dots with a prominent band-edge photoluminescence were obtained by a soft AOT water-in-oil (w/o) microemulsion templating method with an estimated size of 2.7 nm. The CdSe particles were covered with a TiO_2 _layer using an intermediate SiO_2 _coupling reagent by a sol-gel process. The resulting CdSe/TiO_2 _core/shell nanoparticles showed appreciable photocatalytic activity at λ = 405 nm which can only originate because of electron injection from the conduction band of CdSe to that of TiO_2_.

## Introduction

Over the last decade, nanostructured semiconductor materials have been the focus of intense research efforts [1]. The striking feature of a nanometric solid is that conventionally detectable properties are no longer constant, but are tuneable by simply controlling its shape and size, and this has originated a revolution in materials science and device technology. Their photophysics shows high luminescence with tuneable emission maxima and narrow bandwidth. Semiconductor nanocrystals (CdSe, ZnS, etc.), metallic nanocrystals (Ag, Au, etc.) and magnetic nanocrystals (Ni, Fe_3_O_4_, etc.) can be prepared by templating with the aqueous cavities existent in self-organized structures of water-in-oil (w/o) microemulsions [2]. The main aspects that control the structure of these nanoparticulate systems are the nucleation and growth processes, which are determined by the microemulsions dynamics, the interaction between nanoparticle surface, and surfactant molecules and, if needed, by the presence of metal-complexing agents. Core-shell nanoparticles (CdSe/ZnS) have also been prepared by templating techniques [2], opening the range of possibilities for tailoring the material to meet the specific needs of application and improving its biocompatibility. In this study, we succeeded in the production of CdSe quantum dots (QDs) with 2.7 nm size being emitted with high quantum yield at 545 nm with a halfwidth of 30 nm using AOT reverse micelles as templates and polyselenide, Se*_n_*^2-^, as the selenium source. We have grown a titanium dioxide shell above the cadmium selenide core. The huge decrease observed in the photoluminescence (PL) quantum yield of the resulting particles indicates the formation of core-shell CdSe/TiO_2 _nanoparticles, which was reported as due to a photoinduced electron transfer from CdSe to TiO_2 _in a linked arrangement [3]. This process can thus capacitate the TiO_2 _outer layer for electron transfer reactions with adsorbed or surrounding molecules. TiO_2 _can originate this photocatalytic process by itself but, due to a high band gap, UV radiation is needed with λ < 387 nm. The advantage of the prepared nanoparticles is the possibility of efficient use of visible light for the same purpose.

## Experimental

### Chemicals

All the solutions were prepared using spectroscopic grade solvents. Selenium powder (99.5%) was obtained from ACROS. Cadmium nitrate tetrahydrate (98%), sodium sulphide (98%), sodium *bis*(2-ethylhexyl) sulfosuccinate (AOT, 99%), hydrazine, 25%(w/w) solution of tetraethylammonium hydroxide in methanol, (3-mercaptopropyl)trimethoxysilane (95%), tetra-*n*-butylorthotitanate were all obtained from Sigma-Aldrich. Titanium dioxide P25 was donated by Degussa. All the reagents were used as received.

### Preparation of CdSe QDs

Two w/o microemulsions are prepared by injecting a given amount of precursor solutions to a 0.2 M solution of AOT in cyclohexane. The injection is preformed under strong vortexing. In one of the two microemulsions, a cadmium nitrate aqueous solution is injected into the AOT solution followed by a 1 M aqueous solution of sodium sulphide. A solution of polyselenide in DMF was chosen as the precursor for the other microemulsion. This was prepared by a procedure reported by Eggert et al. [4], where hydrazine was added as a reduction agent to an appropriate amount of selenium powder dispersed in DMF, combined with 25% solution of tetraethylammonium hydroxide as an organic base. The process is described by the following equation:

from which one can see that the relation between Se and the organic base determines the type of polyselenide that is formed. The resulting homogeneous solution has a dark green colour. For the preparation of the second microemulsion first a given amount of water is injected, then the sodium sulphide solution and finally the polyselenide/DMF solution. The resulting microemulsion solution acquired a very slight rose coloration. The total aqueous volume is similar to that of the first microemulsion. The final concentration of Cd and Se was 2 × 10^-4 ^M. The used molar ratios were Cd/SO_3_^2- ^= 0.1, Se/hydrazine = 0.5, Se/organic base = 1.5, Se/SO_3_^2- ^= 0.1.

**Figure 1:**
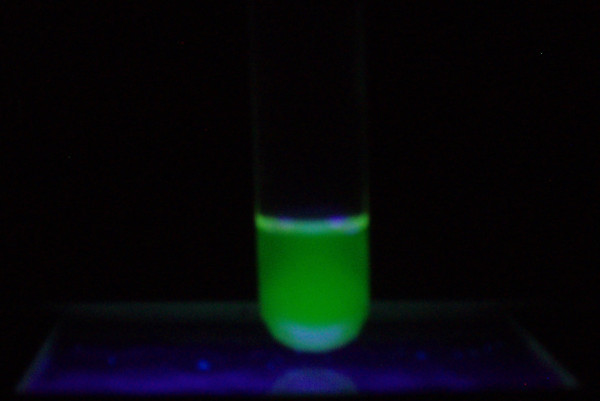
**PL of CdSe QDs under an UV lamp**.

The second microemulsion is added drop by drop to the first one with vortexing. The resulting solution is apparently colourless. After heating at 80°C for 1 h an orange-like colour appears that corresponds to the formation of CdSe QDs. The PL is seen with naked eye using an UV lamp in a dark room (see Figure 1).

### Preparation of CdSe/TiO_2 _nanoparticles

A 1:10 mixture of a (3-mercaptopropyl)trimetoxysilane (MTMS) and tetra-*n*-butylorthotitanate (TBOT) was directly added to the solution of CdSe QDs in AOT. This allowed for the covalent coupling of the QDs surface with silicon alkoxide through its -SH group. The water present in the microemulsion allows for a sol-gel process that results in a small initial layer of SiO_2 _followed by an outer shell of TiO_2_. The solution turned turbid and slightly gelatinous and the fluorescence previously observed for the CdSe QDs disappeared. After heating at 60°C for 45 min, a coloured precipitate settled in the bottom. The colourless supernatant was removed with a pipette, and the solid was washed several times with ethanol to remove the remaining AOT surfactant molecules. The molar ratios used were MTMS/Cd = 1, and TBOT/Cd = 10.

### Spectroscopic measurements

Absorption spectra were recorded using a Shimadzu UV-3101PC UV-Vis-NIR spectrophotometer. Fluorescence measurements were performed using a Fluorolog 3 spectrofluorimeter, equipped with double monochromators in both excitation and emission. Fluorescence spectra were corrected for the instrumental response of the system.

### Irradiation experiments

The irradiation setup is based on a 150-W Xe arc lamp from Lot-Oriel with appropriate interference filters (340 or 405 nm with 10 nm halfwidth) placed before the cuvette holder. A focusing lens was used so that the cuvette could be placed in focus at a distance of 42.5 cm from the lamp with a spot of 8 mm. The cuvette was filled with a 0.1 g/L dispersion of either TiO_2 _from Degussa or the prepared CdSe/TiO_2 _core/shell nanoparticles in a 1.4 × 10^-5 ^M methylene blue (MB) aqueous solution. The light intensity at the cuvette holder was measured using a handheld power meter model 3803 obtained from New Focus. A value of 2.4 mW was obtained at 405 nm using an interference filter from Edmund Optics (20% peak transmission). From the known profile of the arc Xenon lamp and the transmission of a 340 nm interference filter, we can calculate the intensities of the lamp as 3.2 × 10^-8 ^Einstein/cm^2 ^s at 405 nm and 6.9 × 10^-9 ^Einstein/cm^2 ^s at 340 nm.

## Results and discussion

### CdSe QDs

**Figure 2:**
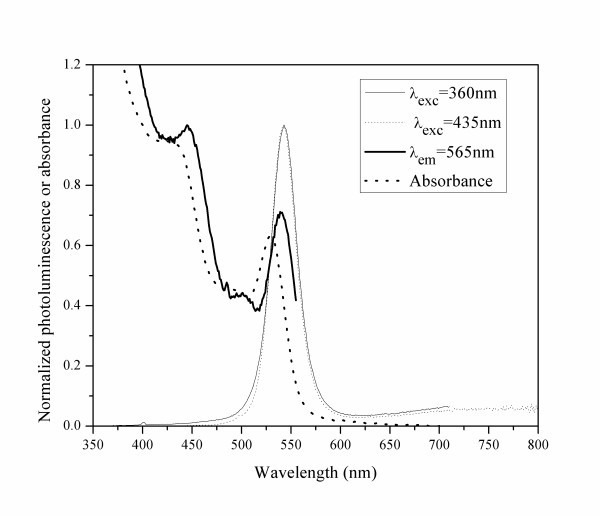
**Absorption and PL spectra of CdSe QDs**.

For the preparation of CdSe QDs, we have used AOT reverse micelles templating procedure, and cadmium nitrate and polyselenide as precursors. The nucleation and growth processes proceed in the water pools, and the resulting particles are probably stabilized by non-covalent surface covering with AOT surfactant molecules. The particle's surface can thus be easily changed, either by adding other molecules that covalently bind to the particles surface displacing the surfactant (capping/functionalization agents), or by growing layers of other materials above the CdSe nanoparticles that can function as nucleation seeds. A more detailed study of the factors that determine the size distribution and quality of the CdSe QDs prepared via polyselenide precursors has been published previously (Fontes Garcia AM, Coutinho PJG: "Production of CdSe Quantum Dots using polyselenide in AOT reverse micelles", submitted). In Figure 2 the absorption, PL and PL excitation (PLE) spectra of CdSe QDs are shown.

Using an empirical relation [5], we can estimate from the first excitonic absorption peak a 2.7 nm particle size. The halfwidth of the PL is about 30 nm, which indicates that the particles are fairly monodisperse although a small red shift of the excitation spectra in relation to the absorption is observed. This comes from the fact that PLE gives the absorption of the subpopulation of particles that contribute more to the emission at the select wavelength. In order to obtain the full range of the absorption spectra, the selected emission wavelength is usually at the red-edge of the PL spectrum. This favours larger particles for which the absorption and PL occur at lower energies (quantum size effect). We thus conclude that the prepared CdSe QDs are not monodisperse but their size distribution is not large on account of the observed small halfwidth of the PL spectrum.

### CdSe/TiO_2 _core-shell nanoparticles

**Figure 3:**
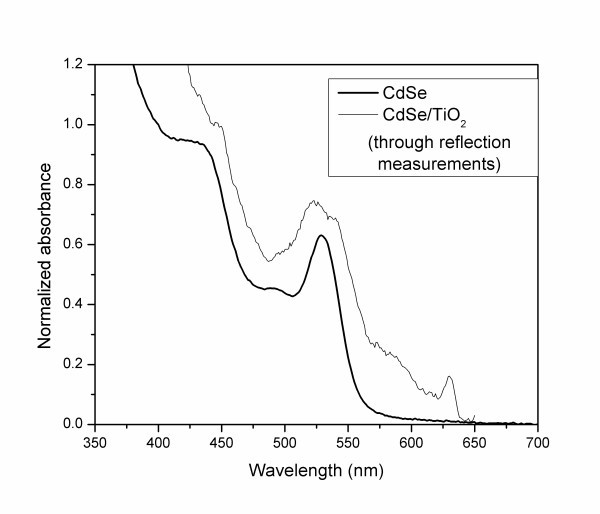
**Absorption spectra of CdSe QDs and CdSe/TiO_2 _core-shell nanoparticles**.

After the addition of the mixture of silicon and titanium alkoxides to the solution of CdSe QDs, the PL disappeared. This indicates that, upon hydrolysis of the alkoxides and covalent coupling through the SH group of MTMS, a mixed layer of SiO_2 _and TiO_2 _is formed above the CdSe nanoparticles. The strong quenching effect observed may be explained by the efficient electron transfer from excited CdSe to TiO_2 _conduction band reported previously [3]. The resulting solution was turbid so that the absorption spectra did not reveal the typical absorption peaks of CdSe. However, by means of reflection measurements of the particles in a capillary, it was possible to obtain the spectrum in Figure 3, which confirms the presence of CdSe nanoparticles with approximately the same size.

### Photodegradation of MB

**Figure 4:**
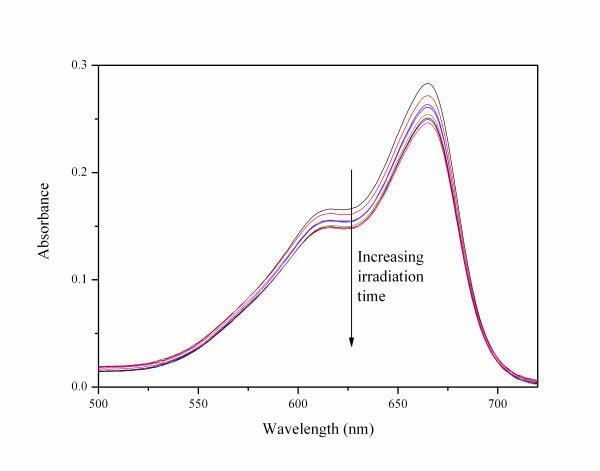
**Photodegradation of MB effected by CdSe/TiO_2 _core-shell nanoparticles at 405 nm**.

**Figure 5:**
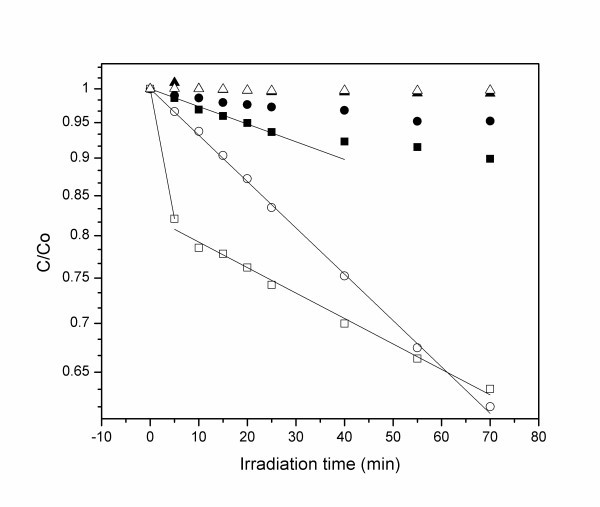
**Photodegradation kinetics of MB using either Degussa TiO_2 _at 340 nm (open circles) and 405 nm (filled circles) or CdSe/TiO_2 _core-shell nanoparticles at 340 nm (open square) and 405 nm (filled square)**. The lines represent first-order exponential kinetics. Control experiments without any photocatalyst at 340 nm (open triangle) and 405 nm (filled triangle) are also shown.

In Figure 4, the photodegradation of MB effected by the prepared CdSe/TiO_2 _core shell nanoparticles is shown. The fraction of the remaining MB in each irradiation time is obtained by subtracting the background from dispersion and comparing the 665 nm absorption peak with the spectrum of pure MB in aqueous solution. The results are shown in Figure 5 for the CdSe/TiO_2 _nanoparticles and for commercial TiO_2 _Degussa (25 nm TiO_2 _nanoparticles) at 340 and 405 nm. The lines represent an exponential decay of MB concentration corresponding to a first-order kinetics. As expected, plain TiO_2 _shows a very inefficient photodegradation rate at 405 nm irradiation. However, at 340 nm, a wavelength well below TiO_2 _band gap, the photodegradation occurs at a rate of 7.0 × 10^-3 ^min^-1^. CdSe/TiO_2 _shows a photodegradation rate of 2.7 × 10^-3 ^min^-1 ^at 405 nm. At 340 nm, a biphasic behaviour occurs at a very fast initial photodegradation rate of 4.0 × 10^-2 ^min^-1 ^followed by slower process at a rate of 3.9 × 10^-3 ^min^-1^. As the TiO_2 _shell cannot absorb blue light, the observed photodegradation process at 405 nm must originate from absorption caused by the CdSe core. This process could be occurring in remaining CdSe QDs that did not couple with TiO_2 _by the sol-gel process [6]. However, the lack of PL contradicts this possibility. On the other hand, if only plain TiO_2 _particles were responsible for the photocatalytic effect, then the dependence of the remaining MB fraction on irradiation time at 340 nm should be similar for Degussa TiO_2 _and CdSe/TiO_2_. This similarity was not observed, as also confirmed in Figure 5, with the photodegradation efficiency of the core-shell nanoparticles being higher than that of Degussa TiO_2_. Thus, we have strong indications that a synergistic effect exists between CdSe and TiO_2 _in the prepared nanoparticles. This effect has been reported in the photoreduction of methyl viologen by CdSe and TiO_2 _nanoparticles confined in the aqueous pools of AOT reversed micelles [7]. A possible mechanism for the photodegradation of MB mediated by CdSe in core-shell CdSe/TiO_2 _involves an electron transfer step from the conduction band of excited CdSe to the conduction band of TiO_2_. This electron may reduce oxygen-generating superoxide anion radical (O_2_^•-^) that in turn may originate OH^• ^radicals. These highly reactive oxygen species can then oxidize MB resulting in its decomposition. The resulting hole in CdSe must be filled to regenerate the catalyst. This can also be accomplished by superoxide radical acting as a reductant and regenerating O_2_.

## Abbreviations

MB: methylene blue; MTMS: (3-mercaptopropyl)trimetoxysilane; PL: photoluminescence; PLE: PL excitation; QDs: quantum dots; TBOT: tetra-*n*-butylorthotitanate.

## Competing interests

The authors declare that they have no competing interests.

## Authors' contributions

PJGC conceived the study, was responsible for its coordination, for the interpretation of results and drafted the manuscript. PJGC was also responsible for the coupling of TiO2 to CdSe QDs. AMFG carried out the CdSe QDs preparation. MSFF carried out the photodegradation measurements. All authors read and approved the final manuscript.
